# Supplementary material for: Molecular rheotaxis directs DNA migration and concentration against a pressure-driven flow
Source: Nat Commun. 2017 Oct 31;8:1213. doi: 10.1038/s41467-017-01214-y (PMC5663963; doi:10.1038/s41467-017-01214-y)
Supplement: Supplementary file 1 — Supplementary Information [file 41467_2017_1214_MOESM1_ESM.pdf]

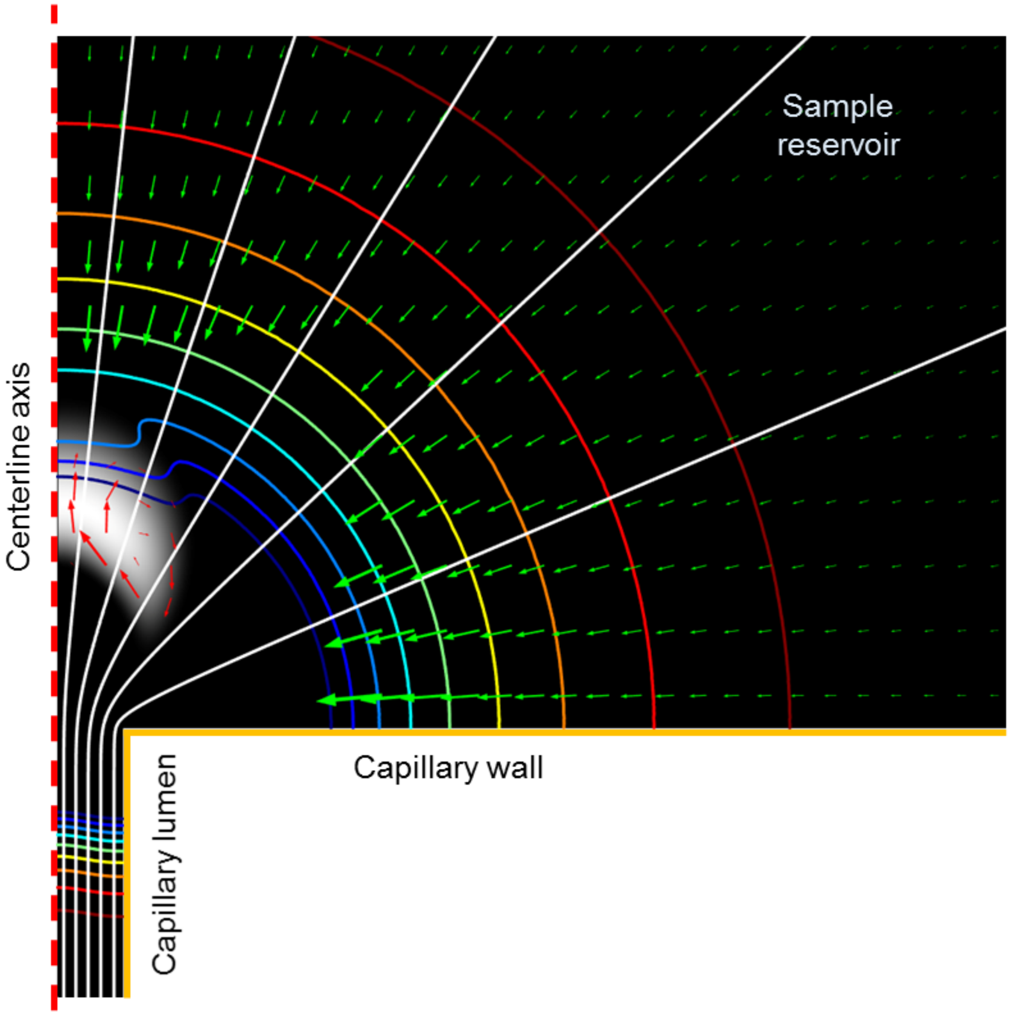

Supplementary Figure 1: Flow streamlines, electric field lines, DNA migration vectors, and DNA concentration heatmap during MRT preconcentration. This image was produced with the simulation and used to generate Fig. 2b within the main text. The dotted red line indicates the centerline of the capillary and the gold lines indicate the walls of the fused silica capillary. The simulation conditions: sample buffer is water and the elution buffer is 2X EB + 18 mM HCl. Counterflow is applied to the sample for 45 minutes at 100 psi. The flow streamlines (white) radiate from the capillary orifice as expected. The electric field (multicolored) also radiates from the capillary orifice but is distorted by the highly concentrated DNA bolus (center). DNA migration is affected by both the electric field and the fluid flow, causing most of the DNA to enter the concentration bolus from near the capillary surface (green arrows). This force balance also causes the recirculating flow pattern of the DNA within the concentrated bolus (red arrows).

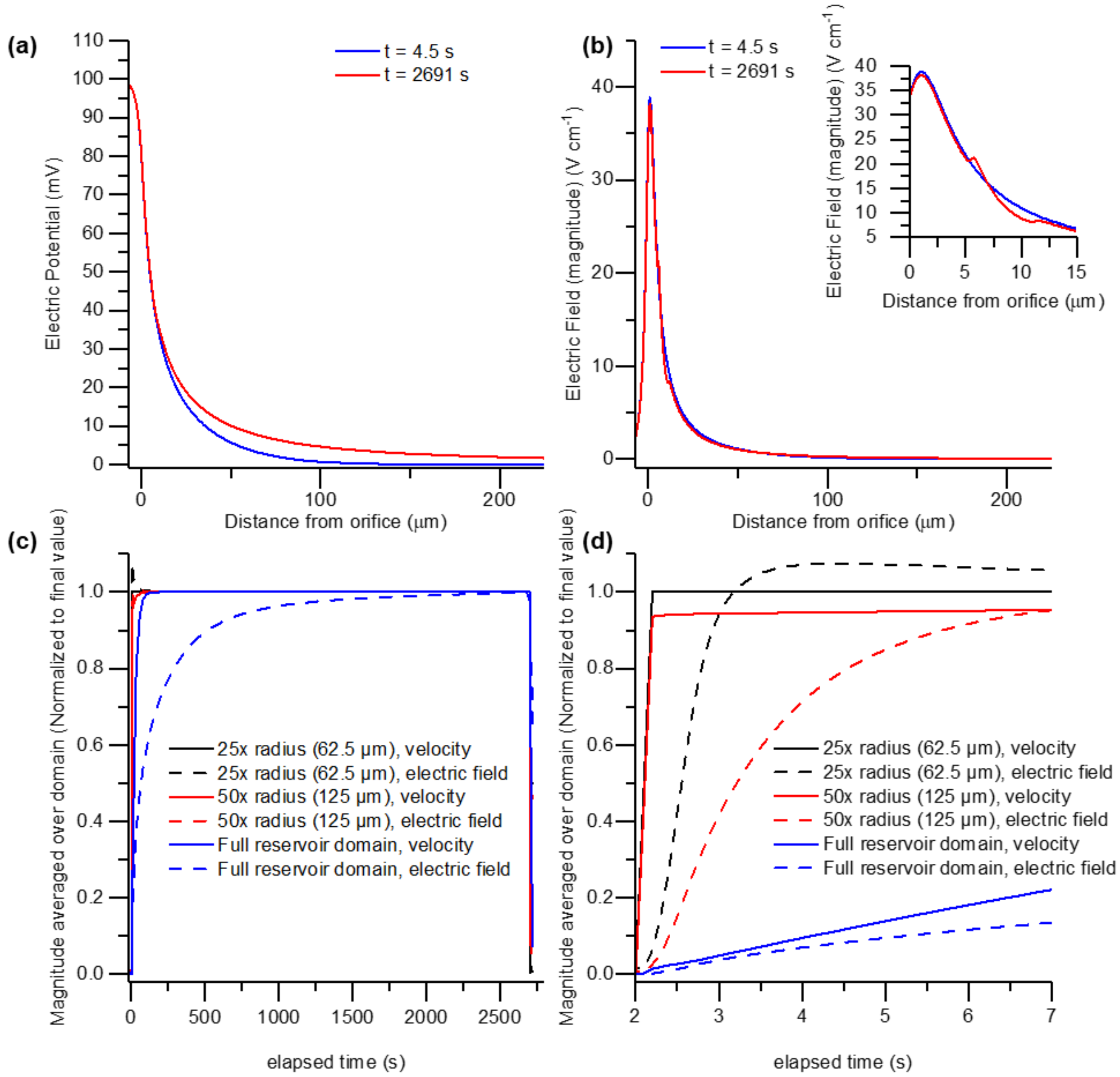

Supplementary Figure 2: Responsivity and stability of fluid flow and induced electric field over 45 minute concentration time. The simulation conditions: sample buffer is water and the elution buffer is 2X EB + 18 mM HCl. Counterflow is applied to the sample for 45 minutes at 100 psi. Plots of the centerline (a) electric potential and (b) electric field at the beginning of fully developed flow (4.5 s), and at a late time point just before injection (2691 s) indicate that the induced electric field establishes quickly and is generally stable with time, with only minor changes observed to both curves over nearly 45 minutes of counterflow. The electric potential extends farther into the reservoir, but the electric field shape does not change significantly with time. The concentrated DNA bolus minorly distorts the electric field near the capillary inlet (inset), but this local field distortion does not affect the global field shape. Domain-averaged magnitude values of the velocity (solid) and electric field (dashed) are plotted over (c) the full concentration period and (d) the

first 5 seconds of concentration. These values were computed over 3 domain sizes: 25x the capillary radius (black), 50x the capillary radius (red), and the full reservoir domain (blue), and normalized to the values at the final time point of applied pressure. Over the full reservoir domain, it takes ~50 s for the fluid flow to stabilize, and the electric field continues to change with time. This is likely due to the low volumetric flow rate (on the order of pL/s) that limits the speed at which the ions can span the full 5 μL reservoir region. However, the bolus concentration region is situated within 25 μm of the capillary orifice (inset of (b)) and is thus contained within a much smaller domain. Within the 62.5 μm domain, the velocity and the electric field stabilize much more quickly: the velocity curve closely follows the imposed pressure ramp rate, and the electric field reaches its maximum with only ~1s lag. The remainder of the reservoir serves as the sink, allowing the field within the bolus region to remain relatively stable.

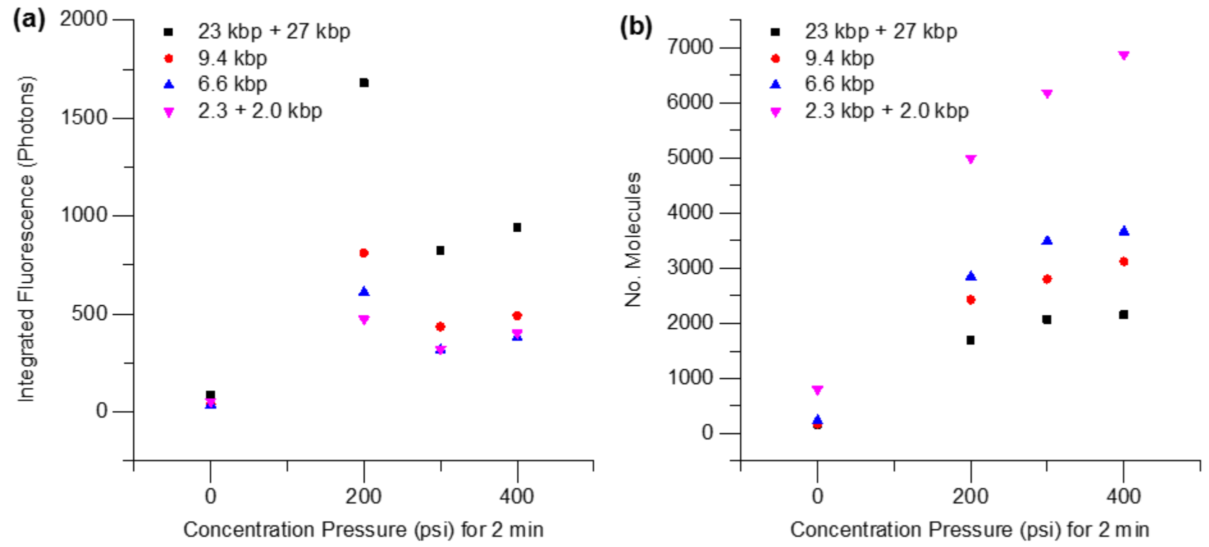

Supplementary Figure 3: Single molecule counting more accurately measures DNA concentration than integration of fluorescence intensity. The sample buffer is 2X EB and the elution buffer is 2X EB + 18 mM HCl. Counterflow is applied to the sample for 2 minutes over a range of counterflow pressures. The raw data traces collected from each separation are then analyzed in terms of both (a) total fluorescence and (b) single molecule counting. Although fluorescence integration analysis suggests that the highest concentration factors are achieved at 200 psi backpressure, single molecule counting reveals that the DNA molecules continue to concentrate at 300 and 400 psi backpressure. This suggests that fluorescence intensity is also affected by the preconcentration technique, and thus integrating fluorescence intensity alone may not accurately represent the actual DNA concentration factors. This is likely due to the salt dependence of intercalating dye binding kinetics[1-3]. For this reason, we did not use fluorescence intensity to calculate concentration factors and limited the experimental conditions to concentrations that allow single molecule counting.

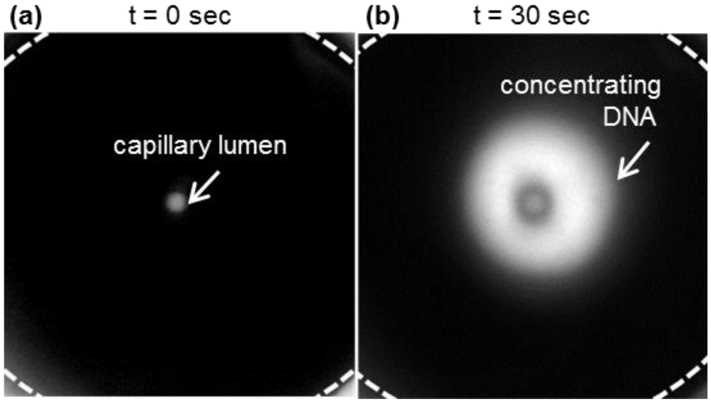

Supplementary Figure 4: At higher flow rates, the concentration bolus forms a doughnut shape surrounding the capillary outlet. These screen shots are taken from Supplementary Movie 4. The microcapillary with a 10 μm lumen diameter and length of 50 cm is filled with 100 mM Tris-HCl buffer. 10 ng/μL *Hind*III digested lambda DNA stained with PicoGreen is surrounding the capillary. 120 psi pressure is applied to the distal capillary end to initiate MRT DNA preconcentration. MRT is not sufficient to overcome the high flow rate exiting the capillary. Instead, the DNA molecules collect around the edges of the capillary lumen, forming a doughnut shaped concentration bolus.

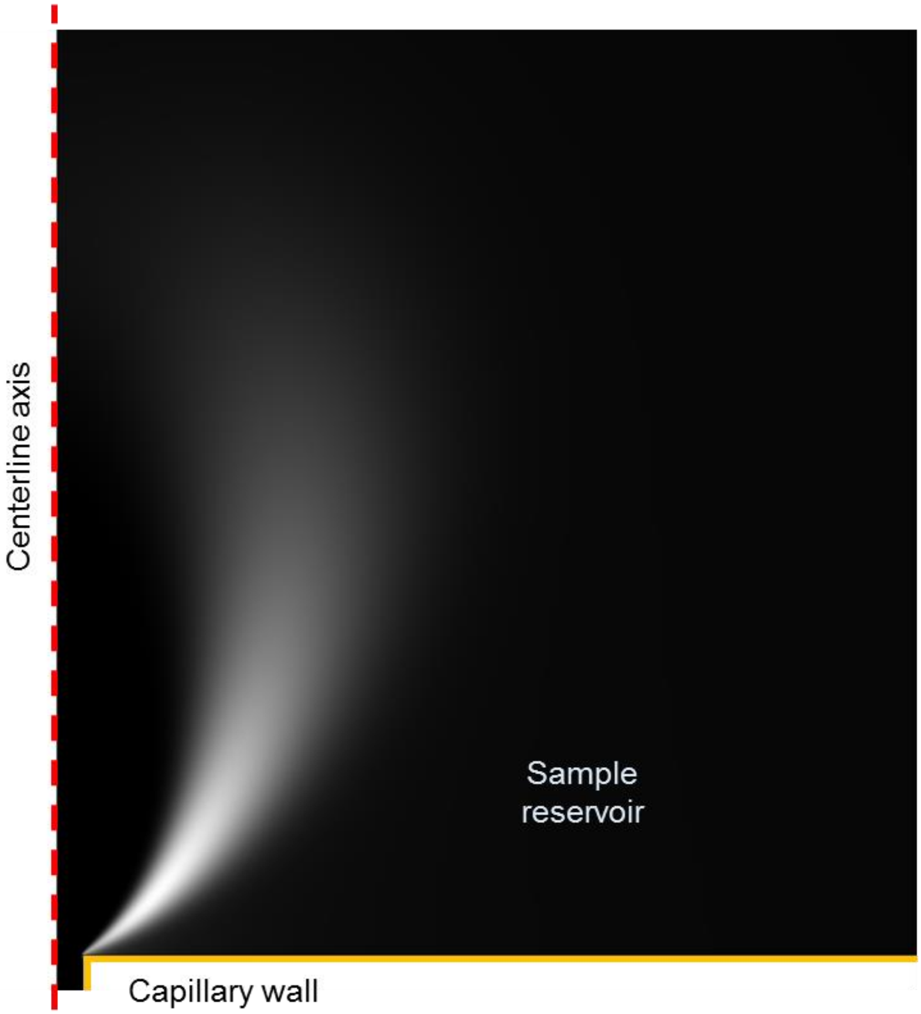

Supplementary Figure 5: DNA concentration profile from 1X EB + 9mM NaCl simulation experiment. The DNA concentration bolus is donut shaped because fluid flow out of the capillary is stronger than the induced electric field. DNA can only concentrate in the slower flow streams away from the capillary center.

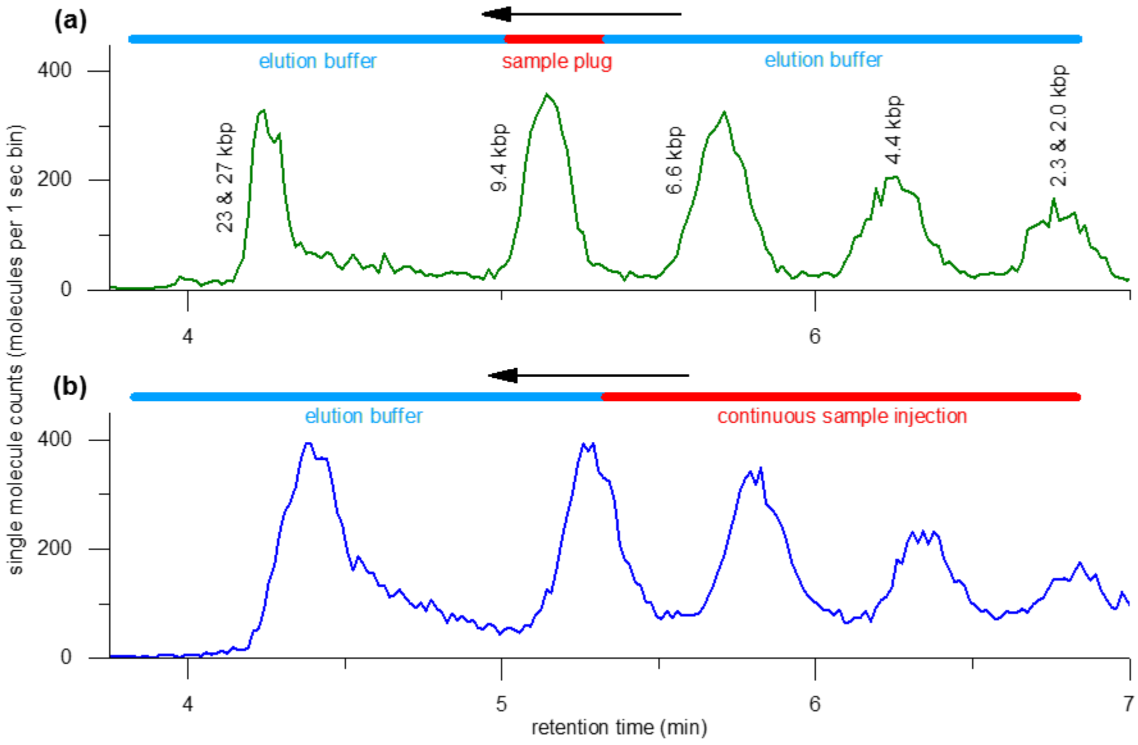

(c)

Fragment Size (kbp)	Number of Molecules		FWHM (min)		Resolution	
	Traditional	1-step	Traditional	1-step	Traditional	1-step
2.0 & 2.3	1744	663	0.21	0.15	1.51	1.75
4.4	2321	1691	0.20	0.17	1.74	1.70
6.6	3183	3272	0.17	0.20	2.06	1.64
9.4	3099	3716	0.14	0.18	4.10	2.48
23 & 27	2207	5105	0.11	0.24		

Supplementary Figure 6: MRT preconcentration enables one-step sample plug injection and separation. Two chromatograms are presented after the same concentration conditions (15 minutes of counterflow at 200 psi). In (a), the traditional 2-step sample plug injection method is employed. The concentrated sample is injected as a plug (10 s injection at 50 psi). Separation is then performed by replacing the sample tube with a buffer tube for separation at 450 psi. In (b), separation is performed directly from the concentrated sample. After concentration, the sample tube is pressurized to 450 psi continuously throughout the entire separation. This allowed full automation from sample concentration through separation and detection without any additional user operation. The table in (c) compares the number of molecules, peak width, and resolution after fitting the chromatograms in (a) and (b).

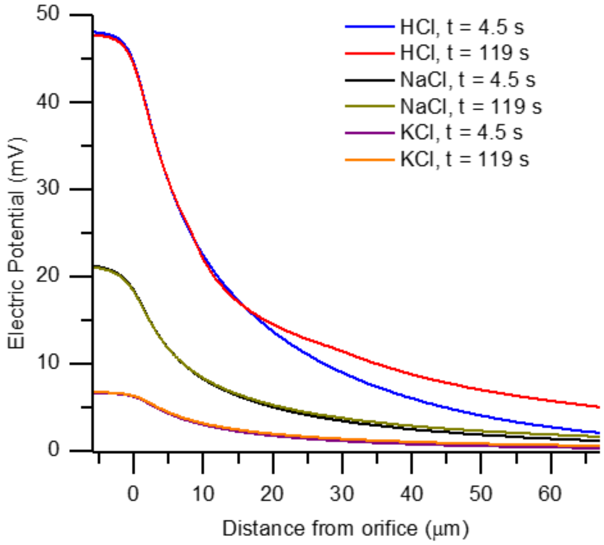

Supplementary Figure 7: Centerline electric potential in simulated experiments with 1X EB + 9mM KCl, HCl, and NaCl running buffers. The smallest potential, KCl, corresponds to the buffer with the majority ions (K^+ and Cl^-) with the most closely matched diffusivities. NaCl generates a larger potential, but the largest potential is generated in HCl.

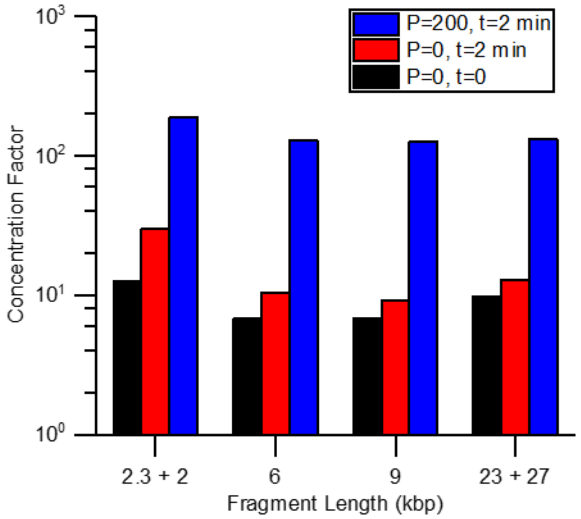

Supplementary Figure 8: Verification that addition of counterflow increases concentration factor for EB Buffer system. The DNA sample is prepared in 2X EB and the running buffer is 2X EB + 18 mM HCl. The minimum achievable time between initial contact between the capillary orifice and sample reservoir and sample injection (limited by the experimental procedure to ~15-30 seconds) is called time t=0. Under these conditions (black), the concentration enhancement is approximately 10-fold. When the capillary outlet and DNA sample reservoir are held in contact for an additional 2 minutes without any applied pressure (red), minimal additional concentration is measured. When 200 psi counterflow is applied to that sample for 2 minutes (blue), the concentration factor increases to 100-fold.

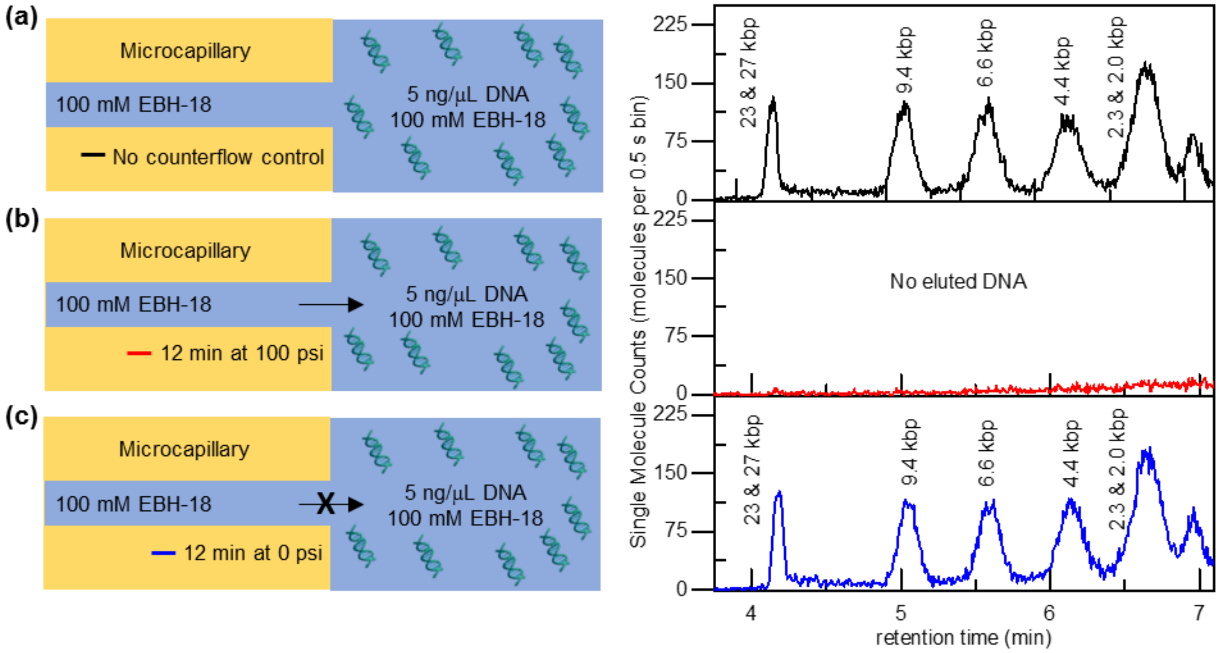

Supplementary Figure 9: Effect of counterflow and DNA diffusion in absence of buffer mismatch. Running buffer and reservoir buffer are 2X EB + 18 mM HCl. *Hind*III digested λ DNA is prepared at 5 ng/mL concentration. (a) Control SML-FSHS separation without counterflow. Following traditional SML-FSHS protocol, the sample plug is injected quickly after the capillary contacts the sample reservoir. The sample reservoir is then exchanged with running buffer, and the sample plug is separated. (b) Counterflow is applied for 12 minutes at 100 psi prior to separation. In the absence of a buffer mismatch, counterflow generated a region of low DNA concentration around the capillary inlet, preventing any DNA molecules from being injected into the capillary when the pressure is reversed. As one would expect, the single molecule chromatogram under these conditions appears empty. (c) The capillary and sample reservoir are held in contact for 12 minutes without a pressure gradient (0 psi) prior to sample injection and separation. The chromatogram is very similar to (a), indicating that flow does not occur in the absence of an applied pressure gradient and that DNA diffusion driven by its own concentration gradient over this time scale is negligeable and cannot be responsible for the high concentration factors achieved in other conditions.

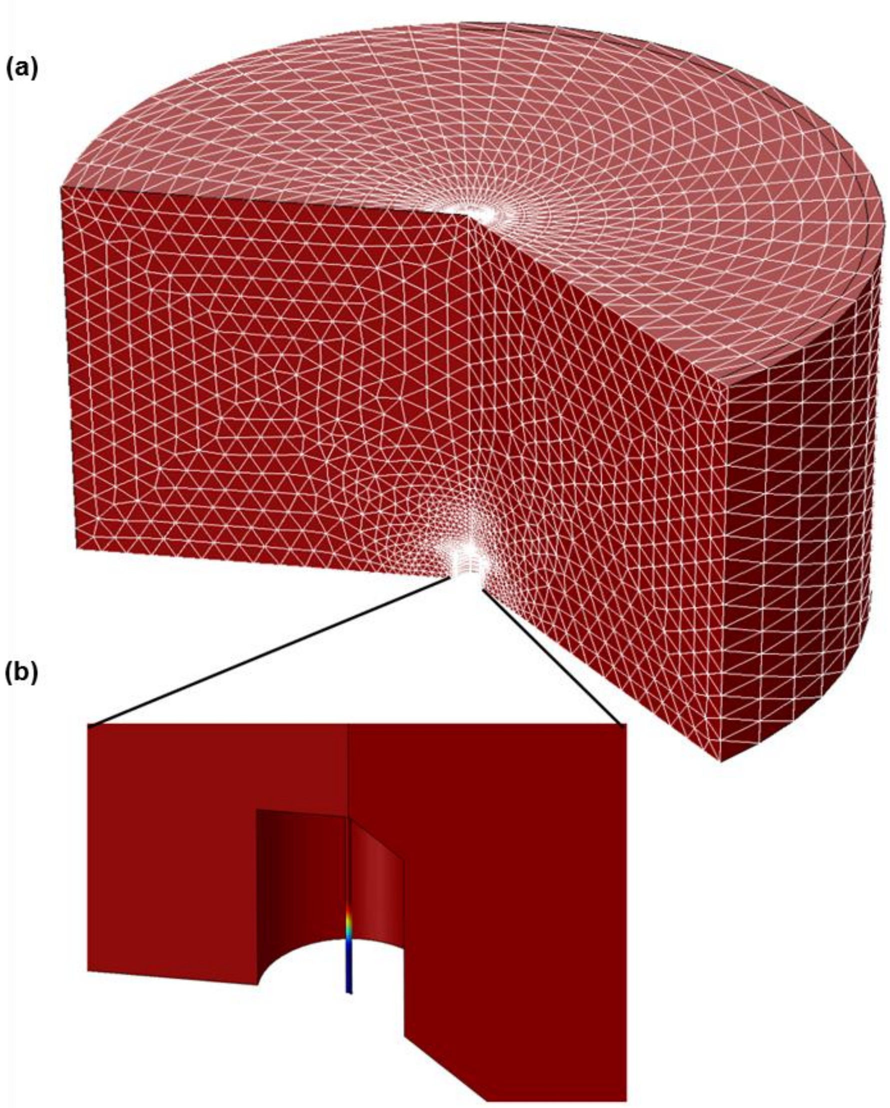

Supplementary Figure 10: Model geometry. (a) The axisymmetric domain used in the COMSOL simulation in this paper consists of a 5 µl reservoir shaped like a right cylinder with the tip of a capillary inserted 150 µm into the reservoir. The mesh size is densest near the capillary orifice and increases in size towards the edges of the reservoir. A very coarse mesh is shown for the purposes of illustration. (b) The inset shows the capillary lumen (the fused silica is not part of the model geometry and is not shown) along with the initial distribution of the dsDNA (red) in the capillary at t=0.

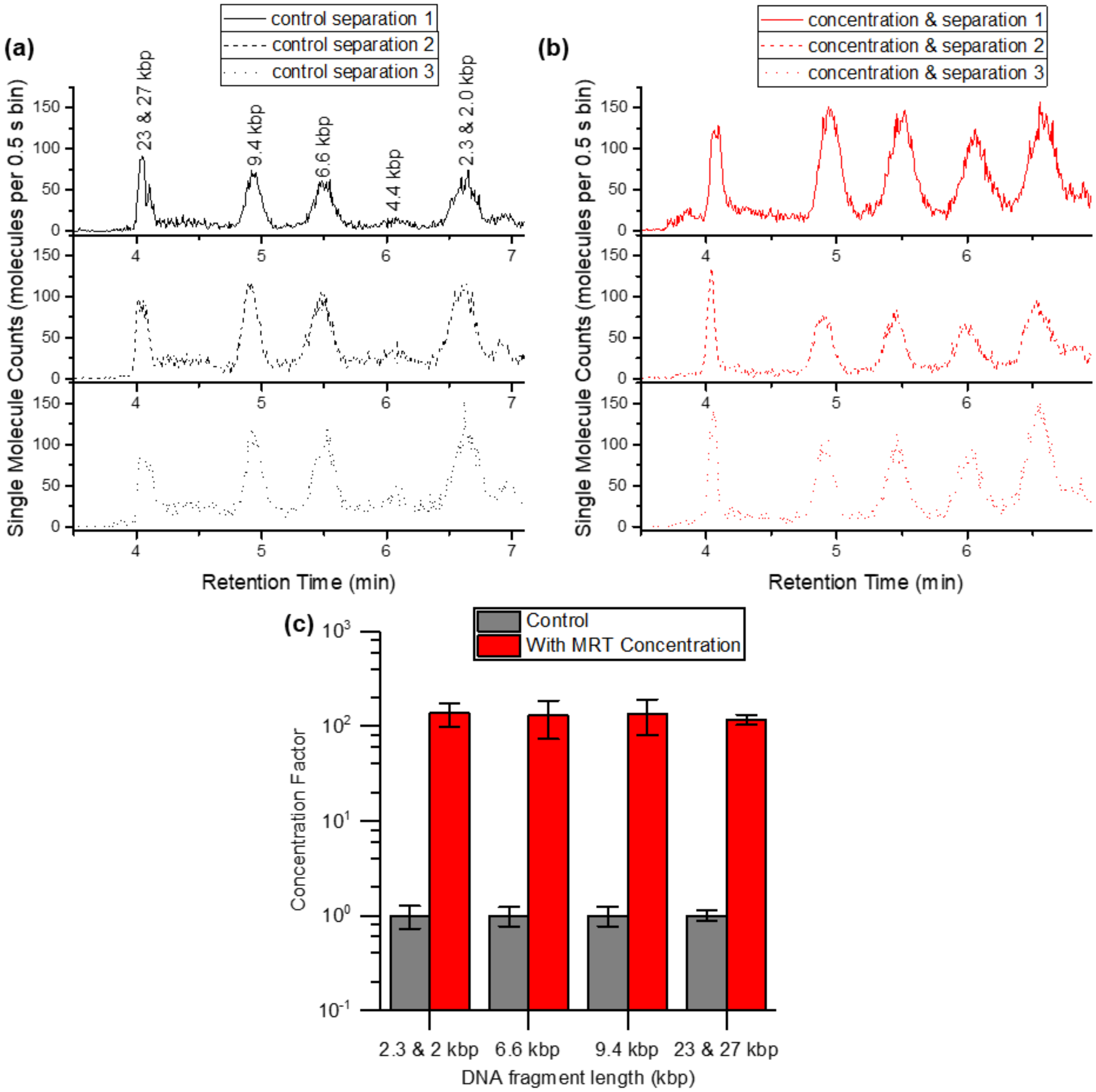

Supplementary Figure 11: Repeatability of SML-FSHS and MRT-SML-FSHS quantification. In (a), SML-FSHS of the same sample is repeated three times. In (b), a 100x diluted sample is concentrated for 2 min at 100 psi before being separated under the same conditions. These chromatograms are analyzed to calculate the average concentration factors for each fragment size, plotted in (c), with error bars representing +/-1 standard deviation. On a single day, coefficient of variation between MRT-SML-FSHS was < 50%, (within an order of magnitude). We did observe larger variation over experiments performed on different days. We attribute this to changes in environmental conditions that we could not control (e.g. temperature, humidity), which appear to affect the concentration enhancement.

Supplementary Table 1: Initial buffer combinations examined to identify MRT mechanism.

	Running Buffer	Reservoir Buffer	Concentration?
1	100 mM Tris-HCl	1 mM Tris-HEPES	Y
2	100 mM Tris-HCl + 0.5% PVP	1 mM Tris-HEPES	Y
3	100 mM Tris-HCl	100 mM Tris-HCl	N
4	1 mM Tris-HEPES	1 mM Tris-HEPES	N
5	1 mM Tris-HEPES	100 mM Tris-HCl	N
6	100 mM Tris-HEPES	1 mM Tris-HEPES	N

Supplementary Table 2: Characteristics of experimental buffers used in main text figures including component concentrations, pH, and principle use.

Buffer Short Name	Buffer Components	pH	Running Buffer	Reservoir Buffer	Figure(s)
100 mM Tris-HCl	100 mM Tris<br>HCl titrated	8.0	X		1
1 mM HEPES-Tris	1 mM HEPES<br>Tris titrated	6.8		X	1
25 mM Tris-HCl	25 mM Tris<br>HCl titrated	8.0	X		3, 4, 8
2X EB + 18 mM HCl	80 mM Bis-Tris<br>20 mM EACA<br>18 mM HCl	7.0	X		2, 7, 8
1X EB+ 9 mM HCl	40 mM Bis-Tris<br>10 mM EACA<br>9 mM HCl	7.0	X		5, 6 (c,f)
1X EB+ 9 mM NaCl	40 mM Bis-Tris<br>10 mM EACA<br>9 mM NaCl	8.0	X		5, 6 (b,e)
1X EB+ 9 mM KCl	40 mM Bis-Tris<br>10 mM EACA<br>9 mM KCl	8.0	X		5, 6 (a,d)
DI Water	--	--		X	2, 3, 4, 7, 8
2X EB	80 mM Bis-Tris<br>20 mM EACA	8.5		X	7
1X EB	40 mM Bis-Tris<br>10 mM EACA	8.5		X	5, 6

Supplementary Note 1: Identification of MRT Mechanism

Based on the migration behavior of the DNA, we postulated that the underlying mechanism was electrokinetic in nature. After ruling out the possibility of spurious electric fields from external power sources, we began to examine mechanisms that would give rise to an internally-generated, local electric field. Given that accumulation of DNA seemed to occur in response to pressure-driven flow from the capillary into the reservoir (Supplemental Movies 1 and 2), we initially hypothesized that a streaming potential was driving the migration. A streaming potential arises when an electrolyte flows through a channel (or capillary) with a charged surface.[4] To elucidate the importance of the streaming potential, we neutralized the negatively charged silica capillary with the addition of polyvinylpyrrolidione (PVP) to the running buffer, a technique commonly used in microfluidic applications to decrease electroosmotic flow (EOF)[5]. If the streaming potential was responsible for the DNA concentration behavior, shielding the charges to reduce the streaming potential would result in a significant decrease or elimination of the concentration effect. Surprisingly, DNA migration and concentration persisted with the addition of 0.5% PVP. This finding was verified with the numerical simulation, in which we observed DNA concentration with both negatively charged and neutrally charged capillary walls. These results suggested that streaming potential does not play a significant role in MRT.

We then examined the importance of the buffer mismatch between the capillary and the reservoir. We first observed the concentration phenomenon when the high ionic strength buffer (100 mM Tris-HCl) filled the capillary and flowed into a low ionic buffer (1mm Tris-HEPES). When we used the same buffer for the running and reservoir buffers, DNA concentration did not occur (Supplementary Table 1, rows 3 and 4). It also did not occur when we reversed the buffers so that the low conductivity buffer flowed into the high conductivity buffer (row 5). Even more surprisingly, DNA concentration did not occur when the capillary was filled with 100 mM Tris-HEPES running buffer and flowed into a reservoir of 1 mM Tris-HEPES (row 6). This suggested that the conditions necessary for DNA concentration to occur were dependent not only on the absolution difference in ionic strength between the capillary and the reservoir, but also on a mismatch in the diffusion coefficient (or mobility) between the anion and cation in the capillary buffer.

Supplementary References

1. Rye HS, Glazer AN. Interaction of dimeric intercalating dyes with single-stranded DNA. *Nucleic Acids Res* **23**, 1215-1222 (1995).

2. Paik DH, Perkins TT. Dynamics and multiple stable binding modes of DNA intercalators revealed by single-molecule force spectroscopy. *Angew Chem Int Ed* **51**, 1811-1815 (2012).

3. Nyberg L, Persson F, Åkerman B, Westerlund F. Heterogeneous staining: A tool for studies of how fluorescent dyes affect the physical properties of DNA. *Nucleic Acids Res* **41**, e184-e184 (2013).

4. Wall S. The history of electrokinetic phenomena. *Current Opinion in Colloid & Interface Science* **15**, 119-124 (2010).

5. Kaniansky D, Masár M, Bielčíková J. Electroosmotic flow suppressing additives for capillary zone electrophoresis in a hydrodynamically closed separation system. *J Chromatogr* **792**, 483-494 (1997).
