# Supplementary material for: Molecular rheotaxis directs DNA migration and concentration against a pressure-driven flow
Source: Nat Commun. 2017 Oct 31;8:1213. doi: 10.1038/s41467-017-01214-y (PMC5663963; doi:10.1038/s41467-017-01214-y)
Supplement: Supplementary file 3 — Description of Additional Supplementary Files [file 41467_2017_1214_MOESM3_ESM.pdf]

Description of Additional Supplementary Files

File Name: Supplementary Movie 1
Description: Concentration of lambda DNA HindIII digest at the orifice of a 5 micrometer inner diameter capillary.

File Name: Supplementary Movie 2
Description: A head on view the orifice of a 10 micrometer inner diameter capillary without applied flow. A concentrated bolus does not grow around the capillary lumen.

File Name: Supplementary Movie 3
Description: Concentration bolus at 1x flow rate (60 psi) in 10 micrometer inner diameter microcapillary.

File Name: Supplementary Movie 4
Description: Concentration bolus forms donut shape at 2x flow rate (120 psi) in 10 micrometer inner diameter microcapillary.

File Name: Supplementary Movie 5
Description: Donut-shaped concentration bolus at 4x flow rate (240 psi) in 10 micrometer inner diameter microcapillary.

File Name: Supplementary Movie 6
Description: High flow rate destabilizes the concentrated DNA bolus, causing it to disengage from capillary orifice.
